# Protein-RNA interface residue prediction using machine learning: an assessment of the state of the art

**DOI:** 10.1186/1471-2105-13-89

**Published:** 2012-05-10

**Authors:** Rasna R Walia, Cornelia Caragea, Benjamin A Lewis, Fadi Towfic, Michael Terribilini, Yasser El-Manzalawy, Drena Dobbs, Vasant Honavar

**Affiliations:** 1 Bioinformatics and Computational Biology Program, Iowa State University, USA; 2 Department of Computer Science, Iowa State University, USA; 3 Center for Computational Intelligence, Learning and Discovery, Iowa State University, USA; 4 College of Information Sciences & Technology, The Pennsylvania State University, University Park, USA; 5 Department of Genetics, Development and Cell Biology, , USA; 6 The Broad Institute, USA; 7 Department of Biology, Elon University, USA; 8 Department of Systems & Computer Engineering, Al-Azhar University, Egypt

## Abstract

**Background:**

RNA molecules play diverse functional and structural roles in cells. They function as messengers for transferring genetic information from DNA to proteins, as the primary genetic material in many viruses, as catalysts (ribozymes) important for protein synthesis and RNA processing, and as essential and ubiquitous regulators of gene expression in living organisms. Many of these functions depend on precisely orchestrated interactions between RNA molecules and specific proteins in cells. Understanding the molecular mechanisms by which proteins recognize and bind RNA is essential for comprehending the functional implications of these interactions, but the recognition ‘code’ that mediates interactions between proteins and RNA is not yet understood. Success in deciphering this code would dramatically impact the development of new therapeutic strategies for intervening in devastating diseases such as AIDS and cancer. Because of the high cost of experimental determination of protein-RNA interfaces, there is an increasing reliance on statistical machine learning methods for training predictors of RNA-binding residues in proteins. However, because of differences in the choice of datasets, performance measures, and data representations used, it has been difficult to obtain an accurate assessment of the current state of the art in protein-RNA interface prediction.

**Results:**

We provide a review of published approaches for predicting RNA-binding residues in proteins and a systematic comparison and critical assessment of protein-RNA interface residue predictors trained using these approaches on three carefully curated non-redundant datasets. We directly compare two widely used machine learning algorithms (Naïve Bayes (NB) and Support Vector Machine (SVM)) using three different data representations in which features are encoded using either sequence- or structure-based windows. Our results show that (i) Sequence-based classifiers that use a position-specific scoring matrix (PSSM)-based representation (PSSMSeq) outperform those that use an amino acid identity based representation (IDSeq) or a smoothed PSSM (SmoPSSMSeq); (ii) Structure-based classifiers that use smoothed PSSM representation (SmoPSSMStr) outperform those that use PSSM (PSSMStr) as well as sequence identity based representation (IDStr). PSSMSeq classifiers, when tested on an independent test set of 44 proteins, achieve performance that is comparable to that of three state-of-the-art structure-based predictors (including those that exploit geometric features) in terms of *Matthews Correlation Coefficient* (MCC), although the structure-based methods achieve substantially higher *Specificity* (albeit at the expense of *Sensitivity*) compared to sequence-based methods. We also find that the expected performance of the classifiers on a residue level can be markedly different from that on a protein level. Our experiments show that the classifiers trained on three different non-redundant protein-RNA interface datasets achieve comparable cross-validation performance. However, we find that the results are significantly affected by differences in the distance threshold used to define interface residues.

**Conclusions:**

Our results demonstrate that protein-RNA interface residue predictors that use a PSSM-based encoding of sequence windows outperform classifiers that use other encodings of sequence windows. While structure-based methods that exploit geometric features can yield significant increases in the *Specificity* of protein-RNA interface residue predictions, such increases are offset by decreases in *Sensitivity*. These results underscore the importance of comparing alternative methods using rigorous statistical procedures, multiple performance measures, and datasets that are constructed based on several alternative definitions of interface residues and redundancy cutoffs as well as including evaluations on independent test sets into the comparisons.

## Background

RNA molecules play important roles in all phases of protein production and processing in the cell [1-4]. They carry the genetic message from DNA to the ribosome, help catalyze the addition of amino acids to a growing peptide chain, and regulate gene expression through miRNA pathways. RNA molecules also serve as the genetic material of many viruses. Many of the key functions of RNA molecules are mediated through their interactions with proteins. These interactions involve sequence-specific recognition and recognition of structural features of the RNA by proteins, as well as non-specific interactions. Consequently, understanding the sequence and structural determinants of protein-RNA interactions is important both for understanding their fundamental roles in biological networks and for developing therapeutic applications.

The most definitive way to verify RNA-binding sites in proteins is to determine the structure of the relevant protein-RNA complex by X-ray crystallography or NMR spectroscopy. Unfortunately, protein-RNA complex structures have proven difficult to solve experimentally. Other methods for determining RNA-binding sites in proteins are costly and time consuming, usually requiring site-directed mutagenesis and low-throughput RNA-binding assays [5-7]. Despite experimental challenges, the number of protein-RNA complexes in the PDB has grown rapidly in recent years (yet still lags behind protein-DNA complexes). As of March 2012, there were 1,186 protein-RNA complexes in the Protein Data Bank [8] and 2,200 protein-DNA complexes.

The difficulties associated with experimental determination of RNA-binding sites in proteins and their biological importance have motivated several computational approaches to these problems [9,10]. Computational methods can rapidly identify the most likely RNA-binding sites, thus focusing experimental efforts to identify them. Ideally, such methods rely on readily available information about the RNA-binding protein, such as its amino acid sequence. Accurate prediction of protein-RNA interactions can contribute to the development of new molecular tools for modifying gene expression, novel therapies for infectious and genetic diseases, and a detailed understanding of molecular mechanisms involved in protein-RNA recognition. In addition to reducing the cost and effort of experimental investigations, computational methods for predicting RNA-binding sites in proteins may provide insights into the recognition code(s) for protein-RNA interactions. Several previous studies have analyzed protein-RNA complexes to define and catalog properties of RNA-binding sites [11-16]. These studies have identified specific interaction patterns between proteins and RNA and suggested sequence and structural features of interfaces that can be exploited in machine learning methods for analyzing and predicting interfacial residues in protein-RNA complexes.

Over the past 5 years, a large number of methods for predicting RNA-binding residues in proteins have been published [12,13,17-34]. In these studies, a variety of sequence, structural, and evolutionary features have been used as input to different machine learning methods such as Naïve Bayes (NB), Support Vector Machine (SVM), and Random Forest (RF) classifiers [9,10]. Most of the methods train classifiers or predictors that accept a set of residues that are sequence or structure neighbors of the target residue as input, and produce, as output, a classification as to whether the target residue is an interface residue. Such methods can be broadly classified into: (i) Sequence-based predictors that encode the target residue and its sequence neighbors in terms of sequence-derived features, e.g., identities of the amino acids, dipeptide frequencies, position-specific scoring matrices (PSSMs) generated by aligning the sequence with its homologs, or physico-chemical properties of amino acids; (ii) Structure-based predictors that encode the target residue and its spatial neighbors in terms of structure-derived features, e.g., parameters that describe the local surface shape; and (iii) Methods that use both sequence- and structure-derived features. The predictors not only differ in terms of the specific choice of the sequence- or structure-derived features used for encoding the input, but also in the methods used (if any) to select a subset of features from a larger set of candidate features, and the specific machine learning algorithms used to train the classifiers, e.g., NB, SVM, and RF classifiers.

Identifying the relative strengths and weaknesses of the various combinations of machine learning methods and data representations is a necessary prerequisite for developing improved methods. However, because of differences in the criteria used to define protein-RNA interfaces, choice of datasets, performance measures, and data representations used for training and assessing the performance of the resulting predictors, as well as the general lack of access to implementations or even complete detailed descriptions of the methods, it has been difficult for users to compare the results reported by different groups. Consequently, most existing comparisons of alternative approaches, including that of Perez-Cano et al. [9], rely on extrapolations of results obtained using different datasets, experimental protocols, and performance metrics. Implementations of some methods are only accessible in the form of web servers. Recently, Puton et al. [10] presented a review of computational methods for predicting protein-RNA interactions in which they compare the performance of multiple web servers that implement different sequence- and/or structure-based predictors on a dataset of 44 RNA-binding proteins (RB44). Such a comparison is valuable for users because it identifies servers that provide more reliable predictions. Use of such servers to compare different methods or data representations can be problematic, however, because it is often impossible to definitively exclude overlap between the training data used for developing the prediction server and the test data used for measuring the performance of the server. In cases where one has access to the code used to generate data representations and implement the machine learning methods, it is possible to use statistical cross-validation to obtain rigorous estimates of the comparative performance of the methods. Comparison of performance of alternative methods based on published studies is fraught with problems, because of differences in the details of the evaluation procedures. For example, some studies use sequence-based cross-validation [35] where, on each cross-validation run, the predictor is trained on a subset of the protein sequences and tested on the remaining sequences. Other studies use window-based cross-validation, where sequence windows extracted from the dataset are partitioned into training and test sets used in cross-validation runs. Still others report the performance of classifiers on independent (blind) test sets. Window-based cross-validation has been shown to yield overly optimistic assessments of predictor performance because it does not guarantee that the training and test sequences are disjoint [36]. Even when sequence-based cross-validation is used, the performance estimates can be biased by the degree of sequence identity shared among proteins included in the dataset. The lower the percentage of sequence identity, i.e., redundancy, allowed within the datasets, the smaller the number of sequences in the datasets and the harder the prediction problem becomes. While some studies have used reduced-redundancy datasets, others have reported performance on highly redundant datasets. Taken together, all of these factors have made it difficult for the scientific community to understand the relative strengths and weaknesses of the different methods and to obtain an accurate assessment of the current state of the art in protein-RNA interface prediction.

Against this background, this paper presents a direct comparison of sequence-based and structure-based classifiers for predicting protein-RNA interface residues, using several alternative data representations and trained and tested on three carefully curated benchmark datasets, using two widely used machine learning algorithms (NB and SVM). We also compare the performance of the best sequence-based classifier with other more complex structure-based classifiers on an independent test set. The goal of this work is to systematically survey some of the most commonly used methods for predicting RNA-binding residues in proteins and to recommend methodology to evaluate machine learning classifiers for the problem. The main emphasis is the evaluation procedure of the different classifiers, i.e., the similarity of protein chains within the datasets used, the way in which cross-validation is carried out (sequence- versus window-based), and the performance metrics reported. Our results suggest that the PSSM-based encoding using amino acid sequence features outperforms other sequence-based methods. In the case of simple structure-based predictors, the best performance is achieved using a smoothed PSSM representation. Interestingly, the performance of the different classifiers was generally invariant across the three non-redundant benchmark datasets (containing 106, 144, and 198 protein-RNA complexes) used in this study. An implementation of the best performing sequence-based predictor is available at http://einstein.cs.iastate.edu/RNABindR/. We also make the datasets available to the community to facilitate direct comparison of alternative machine learning approaches to protein-RNA interface prediction.

### Sequence-based Methods

Early work on the prediction of interface residues in complexes of RNA and protein was carried out by Jeong and Miyano [12], who implemented an artificial neural network that used amino acid type and predicted secondary structure information as input. The dataset used by Jeong and Miyano contained 96 protein chains solved by X-ray crystallography with resolution better than 3Å and was homology-reduced by eliminating sequences that shared greater than 70% similarity over their matched regions. They defined a residue as an interaction residue if the closest distance between the atoms of a protein and its partner RNA was less than 6Å. Terribilini et al. [26] presented RNABindR, which used amino acid sequence identity information to train a Naïve Bayes (NB) classifier to predict RNA-binding residues in proteins. Interface residues were defined using ENTANGLE [37]. They generated and utilized the RB109 dataset (see Methods section) and used sequence-based leave-one-out cross-validation to evaluate classification performance.

Some studies [13,21] have shown that evolutionary information in the form of position-specific scoring matrices (PSSMs) significantly improves prediction performance over single sequence methods. For a given protein sequence, a PSSM gives the likelihood of a particular residue substitution at a specific position based on evolutionary information. PSSM profiles have been successfully used for a variety of prediction tasks, including the prediction of protein secondary structure [38,39], protein solvent accessibility [40,41], protein function [42], disordered regions in proteins [43], and DNA-binding sites in proteins [44]. Kumar et al. [21] developed a support vector machine (SVM) model that was trained on 86 RNA-binding protein (RBP) chains and evaluated it using window-based five-fold cross-validation. This dataset of 86 RBPs contained no two chains with more than 70% sequence similarity with one another. A distance-based cutoff of 6Å was used to define interacting residues. Multiple sequence alignments in the form of PSSM profiles were used as input to the SVM classifier. Kumar et al. were able to demonstrate a significant increase in the prediction accuracy with the use of PSSMs. Wang et al. [30,45] developed BindN, an SVM classifier that uses physico-chemical properties, such as hydrophobicity, side chain *p*_*Ka*_, and molecular mass, in addition to evolutionary information in the form of PSSMs, to predict RNA-binding residues. They evaluated the performance of their classifier by using PSSMs and several combinations of the physico-chemical properties, and found that an SVM classifier constructed using all features gave the best predictive performance. Their classifier was evaluated using window-based five-fold cross-validation. BindN+ [46] was developed by Wang et al. using PSSMs and several other descriptors of evolutionary information. They used an SVM to build their classifier. The method was evaluated using window-based five-fold cross-validation. Cheng et al. introduced smoothed PSSMs in RNAProB [18] to incorporate context information from neighboring sequence residues. In a smoothed PSSM, the score for the central residue i is calculated by summing the scores of neighboring residues within a specified window size (see Methods section for additional details). Cheng et al. evaluated their SVM classifier using window-based five-fold cross-validation and parameter optimization on the RB86 [21], RB109 [26] and RB107 [45] datasets, all used in previous studies. Wang et al. [29] have recently proposed a method that combines amino acid sequence information, including PSSMs and smoothed PSSMs, with physico-chemical properties and predicted solvent accessibility (ASA) as input to an SVM classifier. They utilized a non-redundant dataset of 77 proteins, derived from the RB86 dataset used by Cheng et al. [18] and Kumar et al. [21], by ensuring that no two protein chains shared a sequence identity of more than 25%. Interface residues were defined as those residues in the protein with at least one atom separated by ≤ 6Å from any atom in the RNA molecule. RISP [27] is an SVM classifier that uses PSSM profiles for predicting RNA-binding residues in proteins. An amino acid was defined as a binding residue if its side chain or backbone atoms fell within a 3.5Å distance cutoff from any atom in the RNA sequence. Tong et al. evaluated their classifier using window-based five-fold cross-validation on the RB147 [47] dataset. ProteRNA [19] is another recent SVM classifier that uses evolutionary information and sequence conservation to classify RNA-binding protein residues. Sequence-based five-fold cross-validation on the RB147 dataset was used to evaluate performance. A study that used PSSM profiles, interface propensity, predicted solvent accessibility, and hydrophobicity as features to train an SVM classifier to predict protein-RNA interface residues was conducted by Spriggs et al. [25]. Their method, PiRaNhA, used a non-redundant dataset of 81 known RNA-binding protein (RBP) sequences. It should be noted that the dataset was only weakly redundancy reduced; protein chains with 70% sequence identity over 90% of the sequence length were included in the dataset. An interface residue was defined as any amino acid residue within 3.9Å of the atoms in the RNA. NAPS [48] is a server which uses sequence-derived features such as amino acid identity, residue charge, and evolutionary information in the form of PSSM profiles to predict residues involved in DNA- or RNA-binding. It uses a modified C4.5 decision tree algorithm. Zhang et al. [34] presented a method that uses sequence, evolutionary conservation (in the form of PSSMs), predicted secondary structure, and predicted relative solvent accessibility as features to train an SVM classifier. Performance was evaluated using sequence-based five-fold cross-validation. This study also analyzed the relationship between the various features used and RNA-binding residues (RBRs).

In summary, the primary differences among the methods listed above are: (i) sequence features used, (ii) classifier used, (iii) interface residue definitions, (iv) number of protein-RNA complexes and redundancy levels in the datasets, and (v) cross-validation technique. Interface residue definitions commonly vary between 3.5Å to 6Å. The datasets constructed range from those which contain protein chains that share no more than 70% sequence identity to more stringent collections which share no more than 25% sequence identity. Cross-validation is either window-based or sequence-based.

### Structure-based Methods

Several structure-based methods for predicting RNA-binding sites in proteins have been proposed in literature. KYG [20] is a structure-based method that uses a set of scores based on the RNA-binding propensity of individual and pairs of surface residues of the protein, used alone or in combination with position-specific multiple sequence profiles. Several of the scores calculated are averages over residues located within a certain distance (structural neighbors). Amino acid residues were predicted to be interacting if the calculated scores were higher than a certain threshold. An interface residue was defined as an amino acid residue with at least one RNA atom within a distance of 7Å. Studies [49,50] have shown that structural properties such as surface geometry (patches and clefts) and the corresponding electrostatic properties, patch size, roughness, and surface accessibility can help to distinguish between RNA-binding proteins (RBPs) and non-RBPs as well as between RNA-binding surfaces and DNA-binding surfaces. Chen and Lim [17] used information from protein structures to determine the geometry of the surface residues, and classified these as either surface patches or clefts. This was done using gas-phase electrostatic energy changes and relative conservation of each residue on the protein surface. After the identification of patches and clefts on the protein surface, residues within these RNA-binding regions were predicted as interface residues if they had relative solvent accessibilities ≥5*%*. OPRA [51] is a method that calculates patch energy scores for each residue in a protein by considering energy scores of neighboring surface residues. The energy scores are calculated using interface propensity scores weighted by the accessible surface area (ASA) of the residue. Residues with better patch scores are predicted to be RNA-binding. In this study, interface residues were defined as those that had at least one amino acid atom within a distance of 10Å from any RNA atom. Zhao et al. [52] introduced DRNA, a method that simultaneously predicts RBPs and RNA-binding sites. A query protein is structurally aligned with known protein-RNA complexes, and if the similarity score is higher than a certain threshold, then the query is predicted as an RBP. Binding energy is calculated using a DFIRE-based statistical energy function, to improve the discriminative ability to identity RBPs versus non-RBPs. The binding sites are then inferred from the predicted protein-RNA complex structure. Residues are defined as interface residues if a heavy atom in the amino acid is <4.5Å away from any heavy atom of an RNA base.

A number of methods have incorporated structural information along with evolutionary information to predict RNA-binding sites. Maetschke and Yuan presented a method [24] that uses an SVM classifier with a combination of PSSM profiles, solvent accessible surface area (ASA), betweenness centrality, and retention coefficient as input features. Performance was evaluated on the RB106 and RB144 datasets, which are slightly modified versions of the benchmark datasets created by Terribilini et al. [26,47]. In the Maetschke and Yuan study, an interface residue is defined using a distance cutoff of 5Å. PRINTR [31] is another method that uses SVMs and PSSMs to identify RNA-binding residues. The method was trained on the RB109 dataset using window-based seven-fold cross-validation. A combination of sequence and structure derived features was used, and the best performance was obtained by using multiple sequence alignments combined with observed secondary structure and solvent accessibility information. Li et al. [33] built a classifier using multiple linear regression with a combination of features derived from sequence alone, such as the physico-chemical properties of amino acids and PSSMs, and structure derived features, such as actual secondary structure, solvent accessibility, and the amino acid composition of structural neighbors. Their method was evaluated using window-based six-fold cross-validation. A recent method proposed by Ma et al. [23] used an enriched RF classifier with a hybrid set of features that includes secondary structure information, a combination of PSSMs with physico-chemical properties, a polarity-charge correlation, and a hydrophobicity correlation. A dataset of 180 RNA-binding protein sequences was constructed by excluding all protein chains that shared more than 25% sequence identity and proteins with fewer than 10 residues. Residues were defined as interacting based on a distance cutoff of 3.5Å. They tested the performance of their classifier using a window-based nested cross-validation procedure, where an outer cross-validation loop was used for model assessment and an inner loop for model selection. A method that encodes PSSM profiles using information from spatially adjacent residues and uses an SVM classifier as well as an SVM-KNN classifier was proposed by Chen et al. [32]. Interface residues were defined using a distance cutoff of 5Å. The performance of the method was tested using window-based five-fold cross-validation.

Towfic et al. [28] exploited several structural features (e.g., roughness, CX values) and showed that an ensemble of five NB classifiers that combine sequence and structural features performed better than a NB classifier that only used sequence features. They trained their method, Struct-NB, on the RB147 dataset, and used sequence-based five-fold cross-validation. Struct-NB was trained using residues known to be on the surface of the protein. Liu et al. [22] used a Random Forest (RF) [53] classifier to predict RNA-binding residues in proteins by combining interaction propensities with other sequence features and relative accessible surface area derived from the protein structure. They defined a mutual interaction propensity between a residue triplet and a nucleotide, where the target residue is the central residue in the triplet. A dataset of 205 non-homologous RBPs was constructed to evaluate their method. Protein chains with greater than 25% and RNA chains with greater than 60% sequence identity were removed from their initial pool of 1,182 protein-RNA chains.

In summary, the structure-based methods described above differ in terms of the features, classifiers, interface residue definitions, datasets, and cross-validation techniques used. Interface residues are typically defined within a range of 3.5Å all the way up to 10Å. Features used include RNA-binding propensities of surface residues, geometry of surface residues, solvent accessible surface area, and secondary structure, among others.

### Assessment of existing methods on standard datasets

In this study, we follow a direct approach for comparing different machine learning methods for predicting protein-RNA interface residues using a unified experimental setting (i.e., all methods are trained and evaluated on the same training and test sets). Therefore, our approach can address questions such as (i) Which feature representation is most useful for this prediction problem? (ii) How does feature encoding using sequence-based or structure-based windows compare in terms of performance? and (iii) Which machine learning algorithm provides the best predictive performance? In our experiments, we used three non-redundant benchmark datasets (RB106, RB144, and RB198; see Table 1) to compare several classifiers trained to predict RNA-binding sites in proteins using information derived from a protein’s sequence, or its structure. Two versions of the datasets were constructed: (i) Sequence datasets, which contain all the residues in a protein chain, and (ii) Structure datasets, which contain only those residues that have been solved in the protein structure (see Methods section for details). The input to the classifier consists of an encoding of the target residue plus its sequence or spatial (based on the structure) neighbors. Each residue is encoded using either its amino acid identity or its PSSM profile obtained using multiple sequence alignment. In addition to the questions posted above, we also address to what extent (if any) the recent increase in the number of protein-RNA complexes available in Protein Data Bank (PDB) over the past 6 years contributes to improved prediction of RNA-binding residues.

**Table 1:** The number of interface and non-interface residues in the datasets used in this study

	**RB106Seq**	**RB106Str**	**RB144Seq**	**RB144Str**	**RB199Seq**	**RB199Str**
**Non-interface residues**	20,172	19,284	27,509	26,128	45,710	43,045
**Interface residues**	4,534	4,534	6,109	6,109	7,950	7,950

### Assessment of methods on an independent test set

Several studies [17,20,51,52] have incorporated structural information, such as interaction propensities of surface residues, geometry of the protein surface, and electrostatic properties, to predict RNA-binding residues. Because it is more difficult to implement some of these methods from scratch, we utilized an independent test set of 44 RNA-binding proteins [10] to compare our best performing sequence-based method with results obtained by Puton et al. [10] using the following structure-based methods: KYG [20], OPRA [51], and DRNA [52]. We also used information about surface residues to filter the results obtained by our best performing sequence-based method to directly compare this simple structure information with more complex structure-based methods.

## Results and discussion

For a rigorous comparison of classifiers trained to predict RNA-protein interfacial residues, we first used a sequence-based five-fold cross-validation procedure (see Methods). The input for each classifier consists of an encoding of the target residue plus its sequence or spatial neighbors. Each residue is encoded using its amino acid identity, its PSSM profile obtained using multiple sequence alignment, or its smoothed PSSM profile. We refer to the classifiers that rely exclusively on sequence as "sequence-based" and those that use structural information only to identify spatial neighbors as "simple structure-based" to distinguish them from structure-based methods (discussed below) that exploit more complex structure-derived information, such as surface concavity or other geometric features. Here, we considered 6 different encodings (see Table 2). IDSeq and IDStr encode each amino acid and its sequence or structural neighbors, respectively, using the 20-letter amino acid alphabet; PSSMSeq and PSSMStr encode each amino acid and its sequence or structural neighbors respectively using their PSSM profiles; SmoPSSMSeq and SmoPSSMStr encode each amino acid and its sequence or structural neighbors by using a summation of the values of the PSSM profiles of neighboring residues and itself (see Methods section for details).

**Table 2:** Six different encodings used in this work

		**Sequence**	**Smoothed**		**Structure**	**Smoothed**
**Classifier**	**Sequence**	**PSSM**	**Sequence PSSM**	**Structure**	**PSSM**	**Structure PSSM**
**NB**	IDSeq NB	PSSMSeq NB	SmoPSSMSeq NB	IDStr NB	PSSMStr NB	SmoPSSMStr NB
**SVM with Linear**						
**Kernel**	IDSeq LK	PSSMSeq LK	SmoPSSMSeq LK	IDStr LK	PSSMStr LK	SmoPSSMStr LK
**SVM with Radial**						
**Basis Function Kernel**	IDSeq RBFK	PSSMSeq RBFK	SmoPSSMSeq RBFK	IDStr RBFK	PSSMStr RBFK	SmoPSSMStr RBFK

Abbreviations used in this manuscript for the six different encodings implemented in this study. (NB - Naïve Bayes, SVM - Support Vector Machine).

Tables 3,4 and 5 compare performance based on the AUC (Area Under the receiver operating characteristic Curve) of the different feature encodings using three different machine learning classifiers: (i) Naïve Bayes (NB), (ii) Support Vector Machine (SVM) using a linear kernel (polynomial kernel with degree of the polynomial *p*=1), and (iii) SVM using a radial basis function (RBF) kernel, using residue-based evaluation (seeMethods section for details). For each dataset, the rank of each classifier is shown in parentheses. The last row in each table summarizes the average AUC and rank for each classifier. Table 3 shows a comparison of the AUC of the different sequence-based methods across the three different sequence datasets (RB106Seq, RB144Seq, and RB198Seq). Following the suggestion of Demšar [54], we present average ranks of the classifiers to obtain an overall assessment of how they compare relative to each other. Based on average rank alone, an SVM classifier that uses the RBF kernel and PSSMSeq encoding, which has an average AUC of 0.80, outperforms the other methods. Table 4 shows a comparison of the AUC of the different sequence-based methods on the structure datasets (RB106Str, RB144Str, and RB198Str). The best performance across all three structure datasets by a sequence-based method is achieved by an SVM classifier using the RBF kernel, which obtains an average AUC of 0.81, using the PSSMSeq encoding. A comparison of the simple structure-based methods on the structure datasets is shown in Table 5. The best performing method uses the SmoPSSMStr encoding (with a window size of 3) as input to an SVM classifier constructed with the RBF kernel, achieving an average AUC of 0.80. Tables 6,7 and 8 compare the AUC of the different feature encodings using the three different machine learning classifiers, using protein-based evaluation (See Methods for details). All AUC values obtained using the protein-based evaluation are lower than those obtained using residue-based evaluation. However, the average ranks of the top-performing methods, as calculated using the two evaluation methods, were equivalent. Protein-based evaluation returns lower AUC values than residue-based evaluations because the former is a more stringent measure of the performance of a classifier. These measures are reported as average values over a subset of protein families in the dataset. Poor performance on the more challenging members of the dataset is more apparent in this type of evaluation.

**Table 3:** Residue-based evaluation of Sequence Methods on Sequence Data

	**IDSeq**	**IDSeq**	**IDSeq**	**PSSM Seq**	**PSSM Seq**	**PSSM Seq**	**Smo PSSM**	**Smo PSSM**	**Smo PSSM**
	**NB**	**LK**	**RBFK**	**NB**	**LK**	**RBFK**	**Seq NB**	**Seq LK**	**Seq RBFK**
**RB106Seq**	0.74 (7)	0.72 (9)	0.73 (8)	0.76 (4.5)	0.78 (2.5)	0.80 (1)	0.75 (6)	0.76 (4.5)	0.78 (2.5)
**RB144Seq**	0.73 (7.5)	0.72 (9)	0.73 (7.5)	0.74 (6)	0.79 (2.5)	0.80 (1)	0.75 (5)	0.77 (4)	0.79 (2.5)
**RB198Seq**	0.72 (8)	0.72 (8)	0.72 (8)	0.73 (6)	0.78 (2.5)	0.80 (1)	0.74 (5)	0.77 (4)	0.78 (2.5)
**Average**	**0.73 (7.5)**	**0.72 (8.7)**	**0.73 (7.8)**	**0.74 (5.5)**	**0.78 (2.5)**	**0.80 (1)**	**0.75 (5.3)**	**0.77 (4.2)**	**0.78 (2.5)**

AUC (averaged over five folds) of sequence methods on sequence data using residue-based evaluation. For each dataset, the rank of each classifier is shown in parentheses. Based on average rank, the best sequence method is the SVM classifier that uses the RBF kernel and PSSMSeq as input. (NB - Naïve Bayes, SVM - Support Vector Machine, LK - Linear Kernel, RBFK - Radial Basis Function Kernel).

**Table 4:** Residue-based evaluation of Sequence Methods on Structure Data

	**IDSeq**	**IDSeq**	**IDSeq**	**PSSM Seq**	**PSSM Seq**	**PSSM Seq**	**Smo PSSM**	**Smo PSSM**	**Smo PSSM**
	**NB**	**LK**	**RBFK**	**NB**	**LK**	**RBFK**	**Seq NB**	**Seq LK**	**Seq RBFK**
**RB106Str**	0.74 (7.5)	0.73 (9)	0.74 (7.5)	0.76 (5.5)	0.78 (3)	0.81 (1)	0.76 (5.5)	0.77 (4)	0.79 (2)
**RB144Str**	0.74 (7)	0.73 (9)	0.74 (7)	0.74 (7)	0.79 (2.5)	0.81 (1)	0.75 (5)	0.77 (4)	0.79 (2.5)
**RB198Str**	0.73 (7)	0.73 (7)	0.73 (7)	0.72 (9)	0.78 (3)	0.80 (1)	0.74 (5)	0.77 (4)	0.79 (2)
**Average**	**0.74 (7.2)**	**0.73 (8.3)**	**0.74 (7.2)**	**0.74 (7.2)**	**0.78 (2.8)**	**0.81 (1)**	**0.75 (5.2)**	**0.77 (4)**	**0.79 (2.2)**

AUC (averaged over five folds) of sequence methods on structure data using residue-based evaluation. For each dataset, the rank of each classifier is shown in parentheses. Based on average rank, the best sequence method is the SVM classifier that uses the RBF kernel and PSSMSeq as input. (NB - Naïve Bayes, SVM - Support Vector Machine, LK - Linear Kernel, RBFK - Radial Basis Function Kernel).

**Table 5:** Residue-based evaluation of Structure Methods on Structure Data

	**IDStr**	**IDStr**	**IDStr**	**PSSM**	**PSSM**	**PSSM**	**Smo PSSM**	**Smo PSSM**	**Smo PSSM**
	**NB**	**LK**	**RBFK**	**Str NB**	**Str LK**	**Str RBFK**	**Str NB**	**Str LK**	**Str RBFK**
**RB106Str**	0.76 (3.5)	0.75 (5.5)	0.76 (3.5)	0.71 (8.5)	0.75 (5.5)	0.74 (7)	0.71 (8.5)	0.78 (2)	0.80 (1)
**RB144Str**	0.77 (3.5)	0.76 (5)	0.77 (3.5)	0.71 (8)	0.75 (6)	0.74 (7)	0.70 (9)	0.79 (2)	0.80 (1)
**RB198Str**	0.76 (4)	0.76 (4)	0.76 (4)	0.70 (6.5)	0.74 (6.5)	0.74 (6.5)	0.67 (9)	0.78 (2)	0.79 (1)
**Average**	**0.76 (3.7)**	**0.76 (4.8)**	**0.76 (3.7)**	**0.71 (8.2)**	**0.75 (6)**	**0.74 (6.8)**	**0.69 (8.8)**	**0.78 (2)**	**0.80 (1)**

AUC (averaged over five folds) of structure methods on structure data using residue-based evaluation. Based on average rank, the best structure method across the three datasets is the SVM classifier that uses the RBF kernel and SmoothedPSSMStr (with a window size of 3) as input (NB - Naïve Bayes, SVM - Support Vector Machine, LK - Linear Kernel, RBFK - Radial Basis Function Kernel) The rank of each classifier is shown in parentheses.

**Table 6:** Protein-based evaluation of Sequence Methods on Sequence Data

	**IDSeq**	**IDSeq**	**IDSeq**	**PSSM Seq**	**PSSM Seq**	**PSSM Seq**	**Smo PSSM**	**Smo PSSM**	**Smo PSSM**
	**NB**	**LK**	**RBFK**	**NB**	**LK**	**RBFK**	**Seq NB**	**Seq LK**	**Seq RBFK**
**RB106Seq**	0.69 (6.5)	0.68 (9)	0.69 (6.5)	0.72 (2)	0.71 (3.5)	0.74 (1)	0.69 (6.5)	0.69 (6.5)	0.71 (3.5)
**RB144Seq**	0.68 (7)	0.67 (9)	0.68 (7)	0.71 (4)	0.73 (2)	0.74 (1)	0.68 (7)	0.70 (5)	0.72 (3)
**RB198Seq**	0.68 (6.5)	0.67 (8.5)	0.68 (6.5)	0.69 (5)	0.72 (2.5)	0.74 (1)	0.67 (8.5)	0.70 (4)	0.72 (2.5)
**Average**	**0.68 (6.7)**	**0.67 (8.8)**	**0.68 (6.7)**	**0.71 (3.7)**	**0.72 (2.7)**	**0.74 (1)**	**0.68 (7.3)**	**0.70 (5.2)**	**0.72 (3)**

AUC (averaged over five folds) of sequence methods on sequence data using protein-based evaluation. Based on average rank, the best sequence method is the SVM classifier that uses the RBF kernel and PSSMSeq as input. (NB - Naïve Bayes, SVM - Support Vector Machine, LK - Linear Kernel, RBFK - Radial Basis Function Kernel) The rank of each classifier is shown in parentheses.

**Table 7:** Protein-based evaluation of Sequence Methods on Structure Data

	**IDSeq**	**IDSeq**	**IDSeq**	**PSSM Seq**	**PSSM Seq**	**PSSM Seq**	**Smo PSSM**	**Smo PSSM**	**Smo PSSM**
	**NB**	**LK**	**RBFK**	**NB**	**LK**	**RBFK**	**Seq NB**	**Seq LK**	**Seq RBFK**
**RB106Str**	0.69 (7.5)	0.69 (7.5)	0.70 (5.5)	0.72 (3)	0.72 (3)	0.74 (1)	0.68 (9)	0.70 (5.5)	0.72 (3)
**RB144Str**	0.68 (7)	0.67 (9)	0.68 (7)	0.71 (4)	0.73 (2)	0.74 (1)	0.68 (7)	0.70 (5)	0.72 (3)
**RB198Str**	0.69 (6)	0.68 (8)	0.69 (6)	0.69 (6)	0.72 (2.5)	0.73 (1)	0.67 (9)	0.70 (4)	0.72 (2.5)
**Average**	**0.69 (6.8)**	**0.68 (8.2)**	**0.69 (6.2)**	**0.71 (4.3)**	**0.72 (2.5)**	**0.74 (1)**	**0.68 (8.3)**	**0.70 (4.8)**	**0.72 (2.8)**

AUC (averaged over five folds) of sequence methods on structure data using protein-based evaluation. For each dataset, the rank of each classifier is shown in parentheses. Based on average rank, the best sequence method is the SVM classifier that uses the RBF kernel and PSSMSeq as input. (NB - Naïve Bayes, SVM - Support Vector Machine, LK - Linear Kernel, RBFK - Radial Basis Function Kernel).

**Table 8:** Protein-based evaluation of Structure Methods on Structure Data

	**IDStr**	**IDStr**	**IDStr**	**PSSM Str**	**PSSM Str**	**PSSM Str**	**Smo PSSM**	**Smo PSSM**	**Smo PSSM**
	**NB**	**LK**	**RBFK**	**NB**	**LK**	**RBFK**	**Str NB**	**Str LK**	**Str RBFK**
**RB106Str**	0.72 (3)	0.71 (7)	0.72 (3)	0.71 (7)	0.72 (3)	0.72 (3)	0.69 (9)	0.71 (7)	0.72 (3)
**RB144Str**	0.71 (6.5)	0.71 (6.5)	0.71 (6.5)	0.71 (6.5)	0.72 (3.5)	0.73 (2)	0.68 (9)	0.72 (3.5)	0.74 (1)
**RB198Str**	0.72 (3.5)	0.72 (3.5)	0.72 (3.5)	0.68 (8)	0.72 (3.5)	0.72 (3.5)	0.66 (9)	0.71 (7)	0.72 (3.5)
**Average**	**0.72 (4.3)**	**0.68 (5.7)**	**0.69 (4.3)**	**0.70 (7.2)**	**0.72 (3.3)**	**0.72 (2.8)**	**0.68 (9)**	**0.71 (5.8)**	**0.73 (2.5)**

AUC (averaged over five folds) of structure methods on structure data using residue-based evaluation. Based on average rank, the best structure method across the three datasets is the SVM classifier that uses the RBF kernel and SmoothedPSSMStr (with a window size of 3) as input (NB - Naïve Bayes, SVM - Support Vector Machine, LK - Linear Kernel, RBFK - Radial Basis Function Kernel) The rank of each classifier is shown in parentheses.

**Figure 1:**
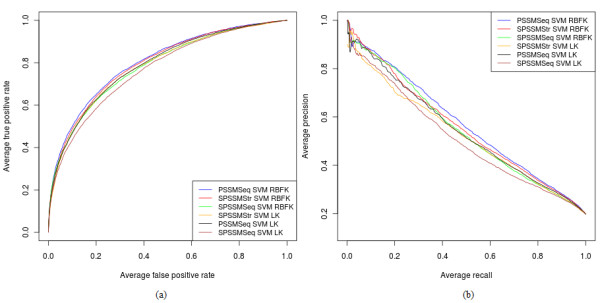
**Top 6 methods on structure datasets.** (**a**) ROC curves and (**b**) PR curves for the top six methods on structure datasets.

Table 9 shows the performance of the 6 top-ranking sequence-based and simple structure-based methods on structure datasets. The feature encoding that gives best performance across all three structure datasets is PSSMSeq, when used as input to an SVM classifier that uses the RBF kernel. Figure 1 shows Receiver Operating Characteristic (ROC) curves and Precision vs Recall (PR) curves for the top performing methods on structure datasets. Notably, classifiers that utilize evolutionary information, i.e., PSSM profiles, have significantly better prediction performance than classifiers that are trained using only the amino acid identities of the target residue and its sequence neighbors.

**Table 9:** Top Six Methods on Structure Data using Residue-Based Evaluation

	**PSSMSeq**	**Smo PSSMSeq**	**PSSMSeq**	**Smo PSSMSeq**	**Smo PSSMStr**	**Smo PSSMStr**
	**RBFK**	**RBFK**	**LK**	**LK**	**RBFK**	**LK**
**RB106Str**	0.81 (1)	0.79 (3)	0.78 (4.5)	0.77 (6)	0.80 (2)	0.78 (4.5)
**RB144Str**	0.81 (1)	0.79 (4)	0.79 (4)	0.77 (6)	0.80 (2)	0.79 (4)
**RB198Str**	0.80 (1)	0.79 (2.5)	0.78 (4.5)	0.77 (6)	0.79 (2.5)	0.78 (4.5)
**Average**	**0.80 (1)**	**0.79 (3.2)**	**0.78 (4.3)**	**0.77 (6)**	**0.80 (2.2)**	**0.78 (4.3)**

Comparison of AUC (averaged over five folds) of the top six methods on structure data using residue-based evaluation. Based on average rank, the best method across all three datasets is the SVM classifier that uses the RBF kernel and PSSMSeq as input. (NB - Naïve Bayes, SVM - Support Vector Machine, LK - Linear Kernel, RBFK - Radial Basis Function Kernel) The rank of each classifier is shown in parentheses.

Additional file 1: Table S1 (see Additional File 1) highlights the similarities and differences between methods implemented in this study and existing methods in the field.

## Representations based on sequence versus structural neighbors

The sequence-based classifiers, IDSeq and PSSMSeq, utilize a sliding-window representation to generate subsequences around residues that are contiguous in the protein sequence. In an attempt to capture the structural context for predicting RNA-binding sites, we constructed the IDStr and PSSMStr encodings which use the spatial neighbors (derived from the 3D structure) of an amino acid as input.

**Figure 2:**
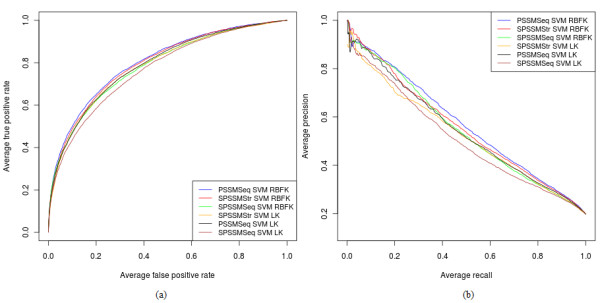
**Naïve Bayes (NB) classifier on the IDSeq and IDStr features using the RB144Str dataset.** (**a**) ROC curves and (**b**) PR curves of the NB classifier on the IDSeq and IDStr features. Both curves are generated using the RB144Str dataset.

Comparison of the ROC curves of the IDSeq_NB and IDStr_NB classifiers (Figure 2a) on the structure dataset (RB144Str) shows that the performance of the IDStr_NB classifier is superior to that of the IDSeq_NB classifier. Similarly, the PR curve (Figure 2b) shows that the IDStr_NB classifier achieves a higher precision at any given level of recall than the IDSeq_NB classifier. The AUC, a good overall measure of classifier performance, is 0.77 for the IDStr_NB classifier compared to 0.74 for the IDSeq_NB classifier on the RB144Str dataset using a Naïve Bayes classifier. The use of spatial neighbors to encode amino acid identity effectively captures information about residues that are close together in the protein structure. It is possible that this encoding indirectly incorporates surface patch information, which leads to improved performance using the IDStr feature, for any choice of classifier.

**Figure 3:**
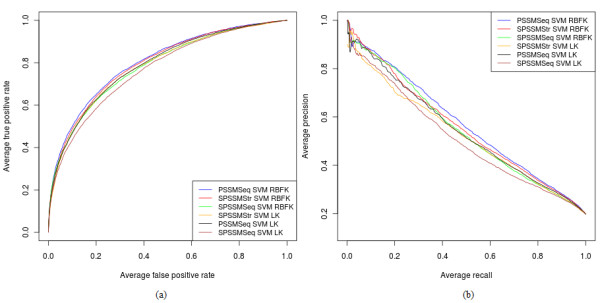
**Support Vector Machine (SVM) classifier with radial basis function (RBF) kernel on the PSSMSeq and PSSMStr features using the RB144Str dataset.** (**a**) ROC curves and (**b**) PR curves of the SVM classifier with an RBF kernel on the PSSMSeq and PSSMStr features. Both curves are generated using the RB144Str dataset.

It is interesting and somewhat surprising that the AUC for the PSSMStr_NB classifier is 0.71, which is lower than 0.74 of the PSSMSeq_NB classifier. This is possibly due to the fact that evolutionary information is encoded linearly in sequence. The use of sequence windows preserves such information while the use of spatial windows does not. Figure 3 shows ROC curves and PR curves for the PSSMSeq_RBF and PSSMStr_RBF SVM classifiers with a radial basis function (RBF) kernel on the RB144Str dataset. The ROC curve for the PSSMSeq_RBF classifier dominates that of the PSSMStr_RBF classifier at all possible classification thresholds. The PR curve also shows that the PSSMSeq_RBF classifier achieves a higher precision for any given level of recall than the PSSMStr_RBF classifier. Similar results were seen for all classifiers on the IDSeq, IDStr, PSSMSeq, and PSSMStr features (see Tables 4 and 5).

## PSSM profile-based encoding of a target residue and its sequence neighbors improves the prediction of RNA-binding residues

Sequence conservation is correlated with functionally and/or structurally important residues [55-57]. We incorporated information regarding sequence conservation of amino acids in our classifiers by using position-specific scoring matrix (PSSM) profiles. PSSMs have been shown to improve prediction performance in a number of tasks including protein-protein interaction site prediction [58], protein-DNA interaction site prediction [44,59], and protein secondary structure prediction [38,39]. PSSMs have been previously shown to improve prediction of RNA-binding sites as well [13,20,21,24,27,31].

**Figure 4:**
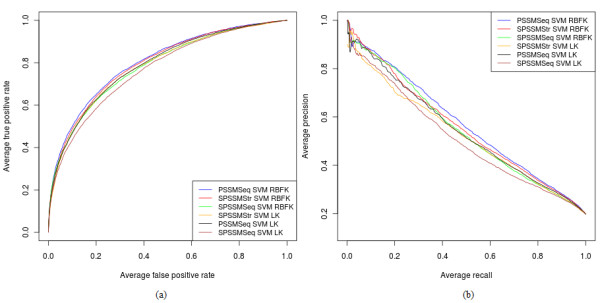
**A comparison of the IDSeq and PSSMSeq features on the RB144Seq dataset.** The PSSMSeq encoding leads to a better prediction performance compared to the IDSeq encoding. (**a**) ROC curves and (**b**) PR curves showing the difference between the IDSeq and PSSMSeq features across 3 different classifiers, Naïve Bayes (NB), Support Vector Machine (SVM) with linear kernel (LK), and SVM with radial basis function (RBF) kernel.

**Figure 5:**
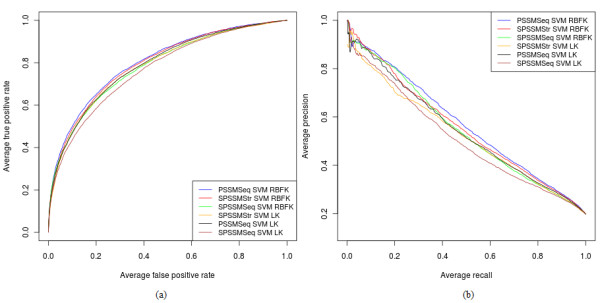
**A comparison of the IDStr and PSSMStr features on the RB144Str dataset.** The IDStr encoding leads to a better prediction performance compared to the PSSMStr encoding. (**a**) ROC curves and (**b**) PR curves showing the difference between the IDStr and PSSMStr features across 3 different classifiers, Naïve Bayes (NB), Support Vector Machine (SVM) with linear kernel (LK), and SVM with radial basis function (RBF) kernel.

In this work, we constructed Naïve Bayes (NB) and Support Vector Machine (SVM) classifiers that utilize PSSM-based encoding of the target residue and its sequence or structural neighbors. The input to the classifiers is a window of PSSM profiles for the target residue and its neighbors in the sequence, in the case of the PSSMSeq classifier, or its spatial neighbors, in the case of the PSSMStr classifier. PSSM-based encoding dramatically improves prediction performance of sequence-based classifiers. Figure 4a shows the ROC curves of the IDSeq and PSSMSeq encodings with three different classifiers on the RB144Seq data. IDSeq_NB has an AUC of 0.73 and PSSMSeq_NB has an AUC of 0.74. The SVM classifier (built using a linear kernel) that used IDSeq has an AUC of 0.72 while the one that used PSSMSeq has an AUC of 0.79. The classifiers that used the PSSMSeq encoding also had a higher specificity for almost all levels of sensitivity (Figure 4b). Evolutionary information, as encoded by PSSMs, does not improve performance in the structure-based classifiers, as shown by the ROC curves in Figure 5a. On the RB144Str data, the SVM classifier built using an RBF kernel has an AUC of 0.74 with PSSMStr, and an AUC of 0.77 with IDStr. Additionally, the precision of the IDStr_RBF encoding is higher for all levels of recall than the PSSMStr_RBF encoding, as shown in Figure 5b.

The main reason that information from multiple sequence alignments improves prediction accuracy is that it captures evolutionary information about proteins. Multiple sequence alignments reveal more information about a sequence in terms of the observed patterns of sequence variability and the locations of insertions and deletions [60]. It is believed that more conserved regions of a protein sequence are either those that are functionally important [61] and/or are buried in the protein core directly influencing the three dimensional structure of the protein and variable sequence regions are considered to be on the surface of the protein [38]. In protein-RNA interactions, RNA-binding residues (RBRs) in proteins play certain functional roles and are thus likely to be moreconserved than non-RBRs. A study by Spriggs and Jones [62] revealed that RBRs are indeed more conserved than other surface residues.

## The predicted solvent accessibility feature does not improve performance of the classifiers

Spriggs et al. [25] combined evolutionary information via PSSMs with the predicted solvent accessibility feature calculated using SABLE [63]. They used an SVM classifier with an RBF kernel and optimized *C* and *γ*parameters to achieve the best AUC values. We performed an experiment to test whether the addition of the predicted solvent accessibility feature (calculated using SABLE with default parameters as in [25]) would improve the performance of our NB classifier and SVM classifier trained using sequence information. This comparative experiment was performed on our smallest dataset, RB106Seq. We combined predicted solvent accessibility with amino acid identity (IDSeq), sequence PSSMs (PSSMSeq), and smoothed PSSMs (SmoPSSMSeq) using a window size of 25. Table 10 shows the average AUC values calculated from a sequence-based five-fold cross-validation experiment. We did not observe any difference in performance by adding the predicted solvent accessibility feature, which is consistent with the study conducted by Spriggs et al. [25] in which they observed a slight improvement after adding predicted solvent accessibility. In the case of the SVM classifier (using an RBF kernel), adding the predicted accessibility feature to the SmoPSSMSeq feature actually led to a decrease in the AUC value. A possible reason for why addition of the predicted solvent accessibility feature did not lead to an improvement in performance for our datasets is that this information is already captured by the other features, such as PSSMs.

**Table 10:** AUC values for different sequence-based features alone and in combination with predicted solvent accessibility

**Features**	**NB**	**SVM RBFK**
**IDSeq**	0.74	0.73
**IDSeq + PA**	0.73	0.73
**PSSMSeq**	0.76	0.80
**PSSMSeq + PA**	0.76	0.80
**SmoPSSMSeq**	0.75	0.78
**SmoPSSMSeq + PA**	0.75	0.75

Comparison of AUC (averaged over five-folds) for different sequence features alone and in combination with predicted solvent accessibility (PA) on the RB106Seq dataset (NB - Naïve Bayes, SVM - Support Vector Machine, RBFK - Radial Basis Function Kernel).

## Classification performance has remained constant as the non-redundant datasets have doubled in size

**Figure 6:**
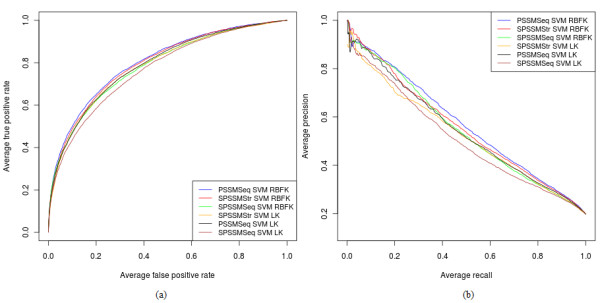
**A comparison of the performance of the Naïve Bayes (NB) classifier on the 3 different sequence datasets using the IDSeq feature.** (**a**) ROC curves and (**b**) PR curves showing the comparison of the performance of the NB classifier using the IDSeq feature on RB106Seq, RB144Seq, and RB198Seq datasets. Prediction performance has not improved as the non-redundant datasets have grown larger.

**Figure 7:**
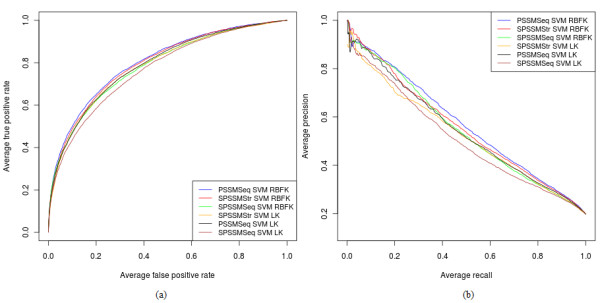
**A comparison of the performance of the Support Vector Machine (SVM) classifier with a radial basis function (RBF) kernel on 3 different structure datasets using the PSSMStr feature.** (**a**) ROC curves and (**b**) PR curves showing the comparison of the performance of the SVM classifier with an RBF kernel using the PSSMStr feature on RB106Str, RB144Str, and RB198Str datasets.

We have attempted to exploit more information about protein-RNA interactions to improve prediction performance by generating a new larger dataset, RB198 (see Methods section), which includes recently solved protein-RNA complexes. The size of the non-redundant dataset has grown from 106 to 198 proteins (as of May 2010), as more complexes have been deposited in the PDB. In this study, we compared three non-redundant datasets, RB106, RB144, and RB198, derived from data extracted from the Protein Data Bank on June 2004, January 2006, and May 2010, using the same exclusion criteria of no more than 30% sequence identity between any two protein chains, and experimental structure resolution of ≤ 3.5Å. To investigate the effect of increasing the size of the non-redundant training set on prediction performance, we trained all of our classifiers on each of the three datasets and compared performance on each. Figure 6 shows the ROC curves for the Naïve Bayes classifier on sequence data for the IDSeq feature. The ROC curves for the RB106Seq, RB144Seq, and RB199Seq datasets, are nearly identical, with AUCs of 0.74, 0.74 and 0.73, respectively. Figure 7 shows the ROC and PR curves for the SVM classifier using an RBF kernel on structure datasets for the PSSMStr feature. These ROC curves are nearly identical, also, with AUCs of 0.74 for all three datasets. The PR curve shows that on the RB198Str dataset, the precision values are actually lower than those obtained using the two smaller datasets, RB106Str and RB144Str, for all values of recall.

Taken together, these results show that the prediction performance as estimated by cross-validation has not improved as the non-redundant dataset has doubled in size. There are several possible explanations for these observations: (i) we have reached the limits of predictability of protein-RNA interface residues using local sequence and simple structural features of interfaces; (ii) the data representations used may not be discriminative enough to yield further improvements in predictions; (iii) the statistical machine learning algorithms used may not be sophisticated enough to extract the information needed to improve the specificity and sensitivity of discrimination of protein-RNA interface residues from non-interface residues; (iv) the coverage of the structure space of protein-RNA interfaces in the available datasets needs to be improved before we can obtain further gains in the performance of the classifiers.

## Comparisons with methods that use more complex structural information

In a recent review, Puton et al. [10] evaluated existing web-based servers for RNA-binding site prediction, including three servers that exploit structure-based information, KYG [20], DRNA [52], and OPRA [51]. To facilitate direct comparison of our results with that study, we evaluated our best sequence-based method, PSSMSeq_RBFK, on the same dataset used in that study. Because our experiments employ a different distance-based interface residue definition (5Å instead of 3.5Å, see Methods), we calculated performance metrics using both definitions. We also calculated both residue-based and protein-based performance measures.

Table 11 shows the performance of different methods on the RB44 dataset using residue-based evaluation. As shown in the table, when evaluated in terms of Matthews Correlation Coefficient (MCC), PSSMSeq_RBFK achieves performance comparable to or slightly lower than that of the structure-based methods: using the 3.5Å interface residue definition (3.5Å IRs), the MCC for PSSMSeq_RBFK is 0.33, whereas the MCC for the structure-based methods ranges from 0.30 - 0.38; using 5.0Å IRs, the MCC for PSSMSeq_RBFK is 0.38 compared with 0.36 - 0.42 for the structure-based methods.

**Table 11:** Residue-based evaluation of Methods on the RB44 Dataset

**Method**	**IR**	**Specificity**	**Sensitivity**	**F measure**	**MCC**
**PSSMSeq_RBFK**	3.5	0.33	0.84	0.47	0.33
	5.0	0.47	0.80	0.59	0.38
**PSSMSeq_RBFK Surface**	3.5	0.36	0.83	0.51	0.37
	5.0	0.51	0.78	0.62	0.42
**KYG**	3.5	0.40	0.73	0.52	0.38
	5.0	0.55	0.67	0.60	0.41
**DRNA **	3.5	0.75	0.27	0.40	0.38
	5.0	0.94	0.23	0.37	0.39
**OPRA **	3.5	0.40	0.54	0.45	0.30
	5.0	0.57	0.51	0.54	0.36

Performance measures computed on the RB44 dataset (IR - Interface Residue definition in Å).

Although the MCC is valuable as a single measure for comparing the performance of different machine learning classifiers, additional performance metrics such as Specificity and Sensitivity can be of greater practical importance for biologists studying protein-RNA interfaces. For example, a high value of Specificity indicates that a prediction method returns fewer false positives, thus allowing biologists to focus on a smaller number of likely interface residues for experimental interrogation. Among all of the methods compared in Table 11, DRNA [52] achieved the highest Specificity values of 0.75 (using 3.5Å IRs) and 0.94 (using 5.0Å IRs). Similarly, when protein-based evaluation is used (Table 12), DRNA achieved the highest Specificity values of 0.94 (using 3.5Å IRs) and 0.98 (using 5.0Å IRs).

**Table 12:** Protein-based evaluation of Methods on the RB44 Dataset

**Method**	**IR**	**Specificity**	**Sensitivity**	**F measure**	**MCC**
**PSSMSeq_RBFK**	3.5	0.32	0.80	0.44	0.27
	5.0	0.45	0.76	0.55	0.30
**PSSMSeq_RBFK Surface**	3.5	0.35	0.79	0.47	0.32
	5.0	0.48	0.74	0.57	0.35
**KYG**	3.5	0.39	0.68	0.49	0.33
	5.0	0.54	0.63	0.56	0.36
**DRNA**	3.5	0.94	0.23	0.21	0.19
	5.0	0.98	0.19	0.21	0.19
**OPRA **	3.5	0.51	0.46	0.36	0.21
	5.0	0.64	0.45	0.43	0.25

The values reported are averages over the 44 proteins in the dataset (IR - Interface Residue definition in Å).

Among classifiers compared using 5.0Å IRs and residue-based evaluation, PSSMSeq_RBFK_Surface returned the best MCC of 0.42. PSSMSeq_RBFK_Surface takes predictions from PSSMSeq_RBFK and considers whether a predicted interface residue is a surface residue or not. If a residue is predicted as an interface residue but is not a surface residue, then it is marked as a non-interface residue (label = ‘0’). On the other hand, if it is a predicted interface residue and is also a surface residue, then the residue remains an interface residue. Surface residues were calculated using NACCESS [64]. In our study, residues that have >5*%* relative accessible surface area (RSA) are defined as surface residues [14]. PSSMSeq_RBFK_Surface achieved Specificity = 0.51 and Sensitivity = 0.78. KYG had similar performance, achieving Specificity = 0.55, Sensitivity = 0.67, and MCC = 0.41. In contrast, when classifiers are compared using 3.5Å IRs and residue-based evaluation, KYG and DRNA have the highest MCC of 0.38, consistent with the results published in Puton et al. [10]. However, PSSMSeq_RBFK has the highest Sensitivity of 0.84 followed by 0.83 for PSSMSeq_RBFK_Surface. Predictors that achieve high values of Sensitivity return fewer false negative values.

When we utilized protein-based evaluation and 5.0Å IRs, KYG returned the best MCC of 0.36. It achieved Specificity = 0.54, Sensitivity = 0.63, and Fmeasure = 0.56. PSSMSeq_RBFK_Surface had similar performance, achieving MCC = 0.35, Specificity = 0.48, Sensitivity = 0.74, and Fmeasure = 0.57. On the other hand, when classifiers are compared using 3.5Å IRs, unlike the case of residue-based evaluation, DRNA does not emerge as a top method. It has low values of MCC = 0.19, Sensitivity = 0.23, and Fmeasure = 0.21. However, it has a Specificity of 0.94. This is because we assign Specificity = 1 in cases where there are zero true positive and false positive predictions (see Performance Measures for more details). The poor performance of DRNA can be explained by the fact that, in 32 out of the 44 proteins in the dataset, DRNA returns zero true positive and zero false positive predictions. In these cases, it returns a few false negative predictions and a much larger number of true negative predictions which result in an Fmeasure of 0 and MCC of 0 for 32 out of 44 proteins which pulls down the average performance over the 44 proteins to values that are considerably lower than their residue-based counterparts.

Taken together, these results indicate that the performance of different methods is affected by the type of evaluation procedure used, i.e., residue-based or protein-based evaluation. Generally, the performance of the sequence-based classifier, PSSMSeq_RBFK, and the simple structure-based classifier, PSSMSeq_RBFK_Surface, is comparable to that of several structure-based methods that exploit more complex structure features, when evaluated based on MCC. They also outperform structure-based methods in terms of Sensitivity, at the cost of Specificity.

An unexpected result of these studies is the finding that the interface residue definition can have a substantial impact on the performance of methods for predicting RNA-binding sites in proteins. For all of the methods compared in Table 11 and Table 12, using a 5Å instead of 3.5Å definition resulted in an increase in MCC, and Specificity, with a decrease in Sensitivity. Moreover, the differences in performance between methods compared using the same interface residue definition, are substantially smaller than the differences in performance obtained for a single method, using different interface residue definitions. Thus, the interface residue definition is an important factor that must be taken into consideration when comparing different methods for predicting RNA-binding residues.

## Conclusions

Studying the interfacial residues of protein-RNA complexes allows biologists to investigate the underlying mechanisms of protein-RNA recognition. Because experimental methods for identifying RNA-binding residues in proteins are, at present, time and labor intensive, reliable computational methods for predicting protein-RNA interface residues are valuable.

In this study, we evaluated different machine learning classifiers and different feature encodings for predicting RNA-binding sites in proteins. We implemented Naïve Bayes and Support Vector Machine classifiers using several sequence and simple structure-based features and evaluated performance using sequence-based *k*-fold cross-validation. Our results from this set of experiments indicate that using PSSM profiles outperforms all other sequence-based methods. This is in agreement with previously published studies [21,25,27,31,45], which demonstrated increased accuracy of prediction of RNA-binding residues by using PSSM profiles. Taken together, these results indicate that determinants of protein-RNA recognition include features that can be effectively captured by amino acid sequence (and sequence conservation) information alone. However, exploiting additional features of structures (e.g., geometry, surface roughness, CX protrusion index, secondary structure, side chain environment) can result in improved performance as suggested in the studies of Liu et al. [22], Ma et al. [23], Towfic et al. [28] and Wang et al. [31]. We observed that the performance of methods utilizing the PSSMSeq feature is comparable to that of three state-of-the-art structure-based methods [20,51,52] in terms of MCC. Nonetheless, structure-based methods achieve higher values of Specificity than methods that rely exclusively on sequence information.

In conclusion, we suggest that for rigorous benchmark comparisons of methods for predicting RNA-binding residues, it is important to consider: (i) the rules used to define interface residues, (ii) the redundancy of datasets used for training, and (iii) the details of evaluation procedures, i.e., cross-validation, performance metrics used, and residue-based versus protein-based evaluation.

Our benchmark datasets and an implementation of the best performing sequence-based method for predicting protein-RNA interface residues are freely accessible at http://einstein.cs.iastate.edu/RNABindR/.

## Methods

### Datasets

We used homology-reduced benchmark datasets for evaluating our classifiers. All three datasets (RB106, RB144 and RB198) used in this study contain protein chains extracted from structures of protein-RNA complexes in the PDB, after exclusion of structures whose resolution is worse than 3.5Å and protein chains that share greater than 30% sequence identity with one or more other protein chains. RB106 and RB144 were derived from RB109 and RB147 [26,47], respectively, by eliminating three chains in each dataset that are shorter than 40 residues [24]. RB199 [65] is a more recently extracted dataset (May 2010) that contains 199 unique protein chains. To be included in the dataset, proteins must include ≥ 40 amino acids and ≥ 3 RNA-binding amino acids and the RNA in the complex must be ≥ 5 nucleotides long. Upon further examination of RB199, it was discovered that one chain, 2RFK_C, had been included erroneously, and so we consider instead the dataset RB198 which does not include that chain. An amino acid residue is considered an interface residue (RNA-binding residue) if it contains at least one atom within 5Å of any atom in the bound RNA.

For all three datasets, we constructed two different versions of the data, referred to as sequence data and structure data. The rationale for creating two different versions of the same dataset was to ensure fair comparison of the sequence and simple structure-based methods. To achieve this, the sequence and structure methods must be evaluated on exactly the same datasets. The sequence data (RB106Seq, RB144Seq, and RB198Seq) consists of all residues in the protein chain, regardless of whether those residues appear in the solved protein structure. On the other hand, the structure data (RB106Str, RB144Str, and RB198Str) includes only those residues that appear in the solved structure of the protein in the PDB. Because of this difference, the total number of residues in the sequence data is greater than the total number of residues in the structure data. Interface residues are labeled with ‘1’ and non-interface residues are labeled ‘0’. Those residues that appear in the sequence only ( i.e., have not been solved in the structure) are labeled as non-interface residues. Table 1 shows the number of interface and non-interface residues for the datasets used in this study.

RB44 [10] is a dataset of 44 RNA-binding proteins released between January 1st and April 28th 2011 from the PDB. No two protein chains in the dataset share greater than 40% sequence identity.

### Data Representation

In this study, we use three different encodings for amino acids. First, amino acid identity (ID) is simply the one letter abbreviation for each of the twenty amino acids. The second encoding is a position-specific scoring matrix (PSSM) vector for each amino acid. For each protein sequence in the dataset, the PSSM is generated by running PSI-BLAST [66] against the NCBI nr database for three iterations with an E-value cutoff of 0.001 for inclusion in the next iteration. The third encoding is the smoothed PSSM [18].

We employ two methods for capturing the context of an amino acid within the protein. First, sequence-based windows are constructed by using a sliding window approach in which the input to the classifier is the target amino acid and the surrounding *n* residues in the protein sequence. This captures the local context of the amino acid within the protein sequence. Second, structure-based windows are designed to capture the structural context of each amino acid, based on spatial neighboring residues in the protein three dimensional structure. We define the distance between two amino acids to be the distance between the centroids of the residues. The structure-based window consists of the target residue and the nearest *n* residues based on this distance measure.

We use both sequence and simple structural features as input to the different classifiers that we have used. Features derived from protein sequence include the amino acid sequence itself (IDSeq), PSSMSeq, the position-specific scoring matrices (PSSMs) and SmoPSSMSeq, the smoothed PSSMs. IDSeq uses a window of 25 contiguous amino acid residues, with 12 residues on either side of the target residue, that is labeled ‘0’ or ‘1’ depending on whether it is a non-interface or interface residue. PSSMSeq encodes evolutionary information about amino acids. The PSSM is an *n*×20 matrix that represents the likelihood of different amino acids occurring at a specific position in the protein sequence, where *n* is the length of the protein sequence. The PSSMs are generated by PSI-BLAST using three iterations and an E-value of 0.001. PSSMSeq also uses a window size of 25 to encode information about the target residue. All individual values in the PSSM are normalized using the logistic function, $y=\frac{1}{1+e^{-x}}$, where *y* is the normalized value and *x* is the original value. Each target residue is represented by 500 (25×20) features. The smoothed PSSM concept (SmoPSSMSeq) was first introduced by Cheng et al. [18] and was shown to perform significantly well in predicting interface residues for the protein-RNA problem. In the construction of a smoothed PSSM, the score for a target residue *i* is obtained by summing up the scores of neighboring residues. The number of scores to be summed up is determined by the size of the smoothing window. For example, if the smoothing window size is 5, then we sum up scores for residues at positions *i*−2 to *i* + 2 to get the score for residue i. We experimented with a smoothing window size of 3, 5 and 7 and obtained the best performance with a smoothing window size of 3 (data not shown).

IDStr, PSSMStr, and SmoPSSMStr are structural features equivalent to the above sequence features. The major difference between structural and sequence features is that contiguous residues for structural features are listed as those residues that are close to each other (in space) within the structure of the protein, regardless of whether they are contiguous in the protein sequence.

### Classifiers

The Naïve Bayes (NB) classifier is based on Bayesian statistics and makes the simplifying assumption that all attributes are independent given the class label. Even though the independence assumption is often violated, NB classifiers have been shown to perform as well as or better than more sophisticated methods for many problems. In this work, we used the NB implementation provided by the Weka machine learning workbench [67].

Let *X* denote the random variable corresponding to the input to the classifier and *C* denote the binary random variable corresponding to the output of the classifier. The NB classifier assigns input x the class label ‘1’ (interface) if:

$$\frac{P(C=1|X=x)}{P(C=0|X=x)}\geq 1$$

and the class label ‘0’ (non-interface) otherwise. Because the inputs are assumed to be independent given the class, using Bayes’ theorem we have:

$$\frac{P(C=1|X=x)}{P(C=0|X=x)}=\frac{P(C=1)\overset{n}{\underset{i=1}{\prod }}P(X_{i}=x|C=1)}{P(C=0)\overset{n}{\underset{i=1}{\prod }}P(X_{i}=x|C=0)}$$

The relevant probabilities are estimated from the training set using the Laplace estimator [35].

The Support Vector Machine (SVM) classifier finds a hyperplane that maximizes the margin of separation between classes in the feature space. When the classes are not linearly separable in the feature space induced by the instance representation, SVM uses a kernel function K to map the instances into a typically high dimensional kernel-induced feature space. It then computes a linear hyperplane that maximizes the separation between classes in the kernel-induced feature space. In practice, when the classes are not perfectly separable in the feature space, it is necessary to allow some of the training samples to be misclassified by the resulting hyperplane. More precisely, the SVM learning algorithm [68,69] finds the parameters w, b, and slack variables _*ξi*_ by solving the following optimization problem:

$$\begin{array}{ll} {\text{Min}}_{w,b,\xi_{i}}\left(\frac{1}{2}w^{T}w\right)+ & C\overset{n}{\underset{i=1}{\sum }}\xi_{i}\text{subject to}\\ & y_{i}(w^{T}\Phi (x_{i})+b)\geq 1-\xi_{i},\xi_{i}\geq 0,\\ & i=1,2,\ldots ,n \end{array}$$

where $w\in {\mathbb{R}}^{d}$ is a weight vector, *b* is a bias and *Φ* is a mapping function. The larger the value of C, the higher the penalty assigned to errors. We use both the polynomial kernel with *p*=1 (Equation 1) and radial basis function (RBF) kernel with *γ*=0.01 (Equation 2) in our study. For our experiments, we used the SVM algorithm implementation (SMO) available in Weka [67]. We used default parameters for the kernels (*p*=1,*γ*=0.01, and *C*=1.0) without any optimization in our experiments.

(1) $$\begin{array}{lll} K(x_{i},x_{j}) & = & {\left(x_{i}.x_{j}+1\right)}^{p}\\ & & \text{where the degree of the polynomial}\;\\ & & \text{p }\text{ is a user-specified parameter} \end{array}$$

(2) $$\begin{array}{ll} K(x_{i},x_{j})= & \text{exp}(-\gamma {∥x_{i}-x_{j}∥}^{2})\\ & \text{where}\gamma \text{ is a training parameter} \end{array}$$

We trained all three classifiers on the different sequence- and structure-based features that we constructed. We balanced the training datasets for the SVM classifiers by employing undersampling of the majority class (i.e., non-interface residues). We also changed nominal attributes (IDSeq and IDStr) to binary attributes using the Weka unsupervised filter *NominalToBinary* for input to the SVM classifier.

### Performance Measures

All the statistics reported in this work are for the positive class (i.e., interface residues). To assess the performance of our classifiers we report the following measures described in Baldi et al. [70]: Receiver Operating Characteristic (ROC) curve, Precision-Recall (PR) curve, Area Under the ROC Curve (AUC), Specificity, Sensitivity, Fmeasure and Matthews Correlation Coefficient (MCC):

$$\text{Specificity}=\frac{\text{TP}}{\text{TP}+\text{FP}}\text{(Precision)}$$

$$\text{Sensitivity}=\frac{\text{TP}}{\text{TP}+\text{FN}}\text{(Recall)}$$

$$F\;\text{measure}=\frac{2\times \text{Precision}\times \text{Recall}}{\text{Precision}+\text{Recall}}$$

$$\text{MCC}=\frac{\text{TP}\times \text{TN}-\text{FP}\times \text{FN}}{\sqrt{\left(\text{TP}+\text{FN})(\text{TP}+\text{FP})(\text{TN}+\text{FP})(\text{TN}+\text{FN}\right)}}$$

We denote true positives by TP, true negatives by TN, false positives by *FP* and false negatives by FN. The measures describe different aspects of classifier performance. Intuitively, Specificity corresponds to the probability that a positive class prediction is correct; Sensitivity corresponds to the probability that the predictor detects the instances of the positive class. Often it is possible to trade off Specificity against Sensitivity. In the extreme case, a predictor that makes 0 positive predictions (TP + FP = 0, and hence TP = 0 and FP = 0) trivially achieves a Specificity of 1. However, such a predictor is useless in practice because it fails to identify any instances of the positive class, and hence has a Sensitivity as well as MCC of 0. An ideal predictor has both Specificity *and* Sensitivity equal to 1 and Fmeasure as well as MCC equal to 1.

The ROC curve plots the proportion of correctly classified positive examples, True Positive Rate (TPR), as a function of the proportion of incorrectly classified negative example, False Positive Rate (FPR), for different classification thresholds. In comparing two different classifiers using ROC curves, for the same FPR, the classifier with higher TPR gives better performance measures. Each point on the ROC curve represents two particular values of TPR and FPR obtained using a classification threshold *θ*. The ROCR package [71] in R was used to generate all ROC curves and PR curves. PR curves give a more informative picture of an algorithm’s performance when dealing with unbalanced datasets [72]. In our case, we have many more negative examples (non-interface residues) than positive examples (interface residues) in the dataset. In PR curves, we plot precision (specificity) as a function of recall (sensitivity or TPR).

To evaluate how effective a classifier is in discriminating between the positive and negative instances, we report the AUC on the test set, which represents the probability of a correct classification [70]. That is, an AUC of 0.5 indicates a random discrimination between positives and negatives (a random classifier), while an AUC of 1.0 indicates a perfect discrimination (an optimal classifier).

The above performance measures are computed based on a sequence-based *k*-fold cross-validation procedure. *k*-fold cross-validation [35] is an evaluation scheme for estimating the generalization accuracy of a predictive algorithm (i.e., the accuracy of the predictive model on the test set). In a single round of sequence-based cross-validation, *m* protein sequences (*m*=*D*/*k*where *D* is the number of sequences in the dataset) are randomly chosen to be in the test set and all the other sequences are used to train the classifier. Sequence-based cross-validation has been shown to be more rigorous than window-based cross-validation [36], because the procedure guarantees that training and test sets are disjoint at the sequence level. Window-based cross-validation has the potential to bias the classifier because portions of the test sequence are used in the training set. In this work, we report the results of sequence-based five-fold cross-validation.

We report our results using two performance evaluation approaches. The first approach, called protein-based evaluation, provides an assessment of the reliability of predicted interfaces in a given protein. The second approach, which we call residue-based evaluation, provides an assessment of the reliability of prediction on a given residue. Let *S* represent the dataset of sequences. We randomly partition S into *k* equal folds $S_{1},\ldots ,S_{k}$. For each run of a cross-validation experiment, *k*−1 folds are used for training the classifier and the remaining fold is used for testing the classifier. Let $S_{i}=(s_{1_{i}},\ldots ,s_{r_{i}})$ represent the test set on the *i*-th run of the cross-validation experiment (r_i_ is the number of sequences in the test set S_i_). In protein-based evaluation, we calculate for each sequence _*sji*_∈_*Si*_ the true positives (*T*_*Pji*_), true negatives (*T*_*Nji*_), false positives (*F*_*Pji*_), and false negatives (*F*_*Nji*_). These values are then used to compute the true positive rate (*TP*_*Rji*_) and false positive rate (*FP*_*Rji*_) for each protein _*sji*_in the test set _*Si*_. The TPR and FPR values for the *i*-th cross-validation run are then obtained as: $\mathrm{TP}R_{i}=\frac{\underset{j}{\sum }\mathrm{TP}R_{\mathrm{ji}}}{r_{i}}$ and $\mathrm{FP}R_{i}=\frac{\underset{j}{\sum }\mathrm{FP}R_{\mathrm{ji}}}{r_{i}}$. We then report the average TPR and FPR of the classifier over *k*-folds as $\mathrm{TP}R_{\mathrm{protein}}=\frac{\underset{i}{\sum }\mathrm{TP}R_{i}}{k}$. Other performance measures for protein-based evaluation are obtained in an analogous fashion. The residue-based measures are estimated as follows: $TP_{i}=\overset{r_{i}}{\underset{j=1}{\sum }}TP_{\mathrm{ji}}$, $TN_{i}=\overset{r_{i}}{\underset{j=1}{\sum }}TN_{\mathrm{ji}}$, $FP_{i}=\overset{r_{i}}{\underset{j=1}{\sum }}FP_{\mathrm{ji}}$ and $FN_{i}=\overset{r_{i}}{\underset{j=1}{\sum }}FN_{\mathrm{ji}}$. These values are then used to calculate TPR_i_ ( $=\frac{TP_{i}}{TP_{i}+FN_{i}}$) and FPR_i_ ( $=\frac{FP_{i}}{FP_{i}+TN_{i}}$) for the *i*-th cross-validation run. We then report the average TPR of the classifier over the *k*-folds as $\mathrm{TP}R_{\mathrm{residue}}=\frac{\underset{i}{\sum }\mathrm{TP}R_{i}}{k}$. Other residue-based performance measures are obtained in an analogous fashion.

### Statistical Analysis

We used the non-parametric statistical test proposed by Demšar [54] to compare the performance of the different prediction methods across the three benchmark datasets, RB106, RB144, and RB198. First we computed the ranks of the different methods for each dataset separately, with the best performing algorithm getting the rank of 1, the second best rank of 2 and so on. Demšar proposes the Friedman test [73] as a non-parametric test that compares the average ranks of the different classifiers. For the results of the Friedman test to be statistically sound, the number of datasets should be greater than 10 and the number of classifiers should be more than 5 [54]. Because we have only three datasets, the Friedman test is not applicable (and thus, was not performed) and we relied on average rank across the three datasets to compare the performance of the different methods. As noted by Demšar, average rank of the classifier provides a fair means of comparing alternative classifiers.

## Competing interests

The authors declare that they have no competing interests.

## Author’s contributions

VH and DD conceived of the study and contributed to experimental design and writing. RW carried out the implementation, experiments, and analysis with assistance from CC, FT and YE-M. MT and BL prepared the datasets used in the study and performed preliminary experiments. RW prepared the initial manuscript. All authors read and approved the manuscript.

## Supplementary Material

Additional file 1 **Table S1.** Supplementary Table S1, which is too large to include in the text. Similarities and Differences of Methods Implemented in this Study with other Methods in the Field. Click here for file
